# Hypertension intracrânienne idiopathique à l’Hôpital général de référence de Niamey (Niger) : une série de huit cas

**DOI:** 10.48327/mtsi.v6i1.2026.692

**Published:** 2026-01-13

**Authors:** Zakaria MAMADOU, Souleymane MAHAMADOU-ANGO, Moussa TOUDOU-DAOUDA, Inoussa DAOUDA BAKO, Moussa ATTAHER, Haoua SIDIBE, Amadou ABDOU-BACHAROU, Fataoulaye SOUMANA, Éric ADEHOSSI

**Affiliations:** 1 Service de neurologie de l’hôpital général de référence, Niamey, Niger; 2 Service de neurochirurgie de l’hôpital national, Niamey, Niger; 3 Service de neurologie de l’hôpital national Amirou Boubacar Diallo, Niamey, Niger; 4 Service d’imagerie médicale de l’hôpital de référence, Niamey, Niger; 5 Service de neurologie de l’hôpital national, Niamey, Niger; 6 Service de médecine interne, hôpital général de référence, Niamey, Niger

**Keywords:** Hypertension intracrânienne idiopathique, Niger, Afrique subsaharienne, Idiopathic intracranial hypertension, Niger, Sub-Saharan Africa

## Abstract

**Introduction:**

L’hypertension intracrânienne idiopathique (HII) survient généralement chez les femmes jeunes et obèses. L’HII est une pathologie rarement décrite dans les pays d’Afrique subsaharienne. Nous rapportons les caractéristiques cliniques et évolutives de patientes nigériennes diagnostiquées avec une HII à Niamey (Niger).

**Matériel et méthodes:**

Dans cette étude, nous avons analysé rétrospectivement les observations de huit patientes nigériennes diagnostiquées avec une HII dans le service de neurologie de l’hôpital général de référence de Niamey entre le 1^er^ juin 2021 et le 31 mai 2023.

**Résultats:**

Toutes les patientes avaient consulté pour des céphalées et une baisse d’acuité visuelle bilatérale d’appa-rition subaiguë. Leur âge médian était de 29,5 ans. Leur indice de masse corporelle médian était de 30,75 kg/m² (intervalle interquartile [IQR] : 26 et 32). L’examen du fond d’œil a révélé un œdème papillaire bilatéral chez toutes les patientes. La pression médiane du liquide céphalorachidien à l’ouverture était de 30 cm H₂O (IQR : 28,35 et 35). L’imagerie cérébrale et vasculaire était normale. Sous acétazolamide, toutes les patientes ont complètement récupéré leur vision avec une normalisation du fond d’œil, et le traitement a été arrêté. Six mois après l’arrêt de l’acétazolamide, une patiente a rechuté, ce qui a nécessité la réintroduction du traitement à long terme.

**Conclusion:**

L’HII est un trouble neurologique peu courant qui touche généralement les femmes jeunes et obèses, mais son incidence augmente en raison de l’épidémie d’obésité. Il s’agit de la première série de cas nigériens d’HII.

## Abstract (en)

**Introduction:**

Idiopathic intracranial hypertension (IIH) typically affects young, obese women. This condition is rarely described in sub-Saharan African countries. This study reports on the clinical and evolutionary characteristics of patients diagnosed with IIH in Niamey, Niger.

**Materials and methods:**

We retrospectively analyzed observations of eight patients diagnosed with IIH in the neurology department of the Niamey General Reference Hospital between June 1, 2021, and May 31, 2023.

**Results:**

All patients presented with headaches and subacute bilateral visual acuity loss. Their median age was 29.5 years. Their median body mass index (BMI) was 30.75 kg/m² (interquartile range [IQR]: 26–32). Fundus examinations revealed bilateral papilledema in all patients. The median cerebrospinal fluid pressure upon opening was 30 cm H₂O (interquartile range [IQR]: 28.35 to 35). Brain and vascular imaging were normal. All patients completely recovered their vision and experienced normalization of the fundus under acetazolamide treatment, after which treatment was discontinued. Six months after discontinuing acetazolamide, one patient relapsed, necessitating the reintroduction of long-term treatment.

**Conclusion:**

Intracranial hypertension (IIH) is a rare neurological disorder that usually affects young, obese women, but its incidence is increasing due to the obesity epidemic. This is the first series of Nigerien cases of IIH.

## Introduction

L’hypertension intracrânienne idiopathique (HII) survient généralement chez les femmes jeunes et obèses [9]. Dans le Minnesota (USA), l’incidence a plus que doublé passant de 1/100 000 en 1990 à 2,4/100 000 en 2014 du fait de l’augmentation du surpoids et de l’obésité [7]. L’HII est une affection rarement décrite dans les pays d’Afrique subsaharienne. À notre connaissance, seuls les cas de deux patients ont été rapportés en Mauritanie, un pays d’Afrique de l’Ouest dont la population est majoritairement subsaharienne [10]. L’HII est définie par des céphalées avec œdème papillaire et pression d’ouverture du liquide céphalorachidien (PO-LCR) >25 cm H_2_0, sans hydrocéphalie ni syndrome de masse cérébrale à l’imagerie, et avec des analyses biochimiques, bactériologiques et anatomopathologiques normales du LCR [5]. Ce travail a pour but de rapporter les caractéristiques cliniques et évolutives de patientes nigériennes diagnostiquées avec une HII dans le service de neurologie de l’hôpital général de référence de Niamey (Niger).

## Méthodologie

L’hôpital général de référence (HGR) de Niamey est l’un des centres de référence tertiaire du Niger dans la prise en charge des pathologies neurologiques. Le service de neurologie, avec son équipe multidisciplinaire (neurologue, neurophysiologiste, neurovasculaire, kinésithérapeute), offre des soins avancés en neurologie générale, épileptologie, neurophysiologie clinique, pathologies cérébrovasculaires, etc.

Nous avons analysé rétrospectivement les observations de patientes nigériennes diagnostiquées avec une HII dans notre hôpital entre le 1er juin 2021 et le 31 mai 2023. Chaque patiente a bénéficié d’une imagerie du parenchyme cérébral (IRM de bas champs : 0,35 Tesla ou TDM de 16 barrettes), et des vaisseaux intracrâniens (veines et artères), d’une étude du liquide céphalorachidien (LCR : cytorachie, protéinorachie, glycorachie, culture et recherche des cellules atypiques) et d’examens de laboratoire à la recherche d’un syndrome inflammatoire biologique, quantification des d-dimères, etc.). Chez chaque patiente, la pression d’ouverture PO-LCR a été mesurée à l’aide d’un manomètre fixé à l’aiguille alors que la patiente était allongée en décubitus latéral gauche. Toutes les patientes incluses dans l’étude répondaient aux critères de l’International Headache Society (IHS) pour l’HII (dernière version : critères ICHD 3B) [5].

Les informations suivantes ont été recueillies dans les dossiers médicaux de chaque patiente : âge, sexe, indice de masse corporelle (IMC), antécédents médicaux, facteurs de risque cardiovasculaire, résultats de l’examen ophtalmologique, résultats de l’imagerie cérébrale et vasculaire, PO-LCR et résultats de l’analyse du LCR.

Cette étude a suivi les principes de la déclaration d’Helsinki. L’approbation d’un comité d’éthique n’a pas été nécessaire car il s’agissait d’une étude de soins de routine et les données ont été collectées de manière anonyme. Toutes les patientes ont donné leur consentement éclairé pour participer à cette étude.

Les caractéristiques des patientes ont été exprimées en pourcentages pour les variables catégorielles et en médianes avec les intervalles interquartiles (IQR) pour les variables continues. Le test de Shapiro-Wilk a été utilisé pour évaluer la normalité de toutes les mesures quantitatives. Les analyses statistiques ont été réalisées à l’aide du logiciel statistique SPSS (IBM SPSS Statistics for Windows, Version 25.0. Armonk, NY : IBM Corp).

## Résultats

**Figure 1:**
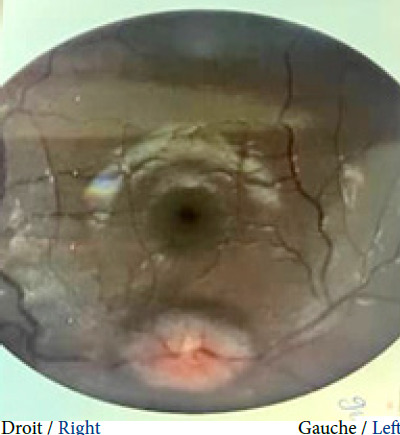
Œdème papillaire

**Figure 2:**
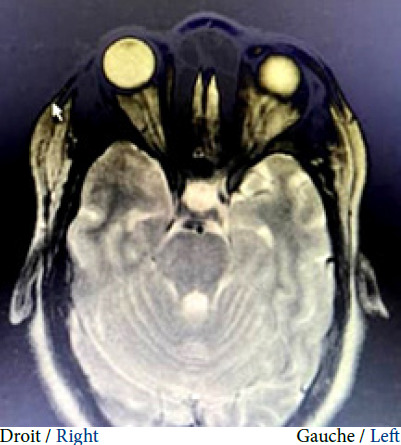
IRM orbito-cérébrale en séquence T2 axiale, montrant une dilatation de la gaine du nerf optique droit

Pendant la période de l’étude, 2 700 patientes ont été vues en consultation, parmi lesquels 8 ont été diagnostiquées avec une HII, soit une prévalence hospitalière de 3 ‰. Le Tableau I détaille les caractéristiques des huit patientes. Toutes les patientes incluses étaient des femmes avec un âge médian de 29,5 ans (IQR : 21,5-37,75) et un IMC médian de 30,75 kg/m² (IQR : 26-32). Elles n’avaient pas d’antécédents médicaux notamment de céphalées dites primaires (migraine, céphalée de tension, etc.) ni de facteurs de risque cardiovasculaire en dehors de l’obésité. Aucune d’entre elles n’était exposée à une pathologie infectieuse notamment à *Mycobacterium tuberculosis*. Six patientes ont été adressées par des ophtalmologues en consultation de neurologie pour des céphalées (d’installation subaiguë) et un œdème papillaire bilatéral. Ce dernier a été observé chez toutes les patientes de notre étude (Fig. 1). L’examen clinique neurologique a révélé une diplopie binoculaire avec paralysie du nerf abducens (nerf VI : oculomoteur externe) chez cinq patientes, le reste de l’examen clinique (général et neurologique) était normal. Sept patientes ont réalisé des IRM cérébrales et une seule a réalisé un scanner cérébral et angioscanner cérébral. L’imagerie cérébrale (parenchyme et vaisseaux) n’a révélé d’anomalie parenchymateuse ou de sténose des sinus latéraux chez aucune patiente. Une dilatation de la gaine du nerf optique (vue en IRM à la séquence T2 coupe axiale et coronale) a été observée chez une patiente (Fig. 2). La pression médiane du LCR à l’ouverture était de 30 cm H_2_O (IQR : 28,35-35). Toutes les patientes ont été traitées avec de l’acétazolamide (250 mg toutes les 8 heures, sur au moins 3 mois avec arrêt dégressif et surveillance de la natrémie et de la kaliémie) et ont récupéré une vision complète avec normalisation du fond d’œil au bout de 2 à 3 mois de traitement, à la suite desquels le traitement a été arrêté. Aucune patiente n’a présenté d’effet secondaire. Le traitement non médicamenteux a consisté à réduire la pression du LCR en soustrayant 40 à 50 ml de LCR lors de la ponction lombaire, et en instaurant un régime hygiéno-diététique amaigrissant. Six mois après l’arrêt de l’acétazolamide, une patiente a récidivé, ce qui a nécessité la réintroduction du traitement à long terme.

**Tableau I:** Caractéristiques clinique et radiologique de 8 cas d’hypertension intracrânienne à l’hôpital général de référence de Niamey (2021-2023)

Variable	Patiente 1	Patiente 2	Patiente 3	Patiente 4	Patiente 5	Patiente 6	Patiente 7	Patiente 8
Âge (ans)	31	28	18	55	23	21	31	40
IMC (kg/m^2^)	28,2	30,2	25,3	32,1	35	31,3	20,2	32
ATCD et FRCV	Non	Non	Non	Non	Non	Non	Non	Non
AV OG	9/10	5/10	8/10	7/10	-	-	4/10	7/10
AV OD	7/10	6/10	8/10	8/10	-	-	5/10	7/10
FO	OPB	OPB	OPB	OPB	OPB	OPB	OPB	OPB
Paralysie du nerf VI	Oui	Non	Oui	Oui	Oui	Non	Oui	Non
SSL	Non	Non	Non	Non	Non	Non	Non	Non
DGNO	Non	Non	Non	Non	Non	Oui	Non	Non
PO-LCR (cmH_2_O)	37	27	35	29	35	28	30	30
Analyse LCR	Normale	Normale	Normale	Normale	Normale	Normale	Normale	Normale

OPB : œdème papillaire bilatéral AV OG : acuité visuelle œil gauche AV OD : acuité visuelle œil droit ATCD : antécédent SSL : sténose du sinus latéral PO-LCR : pression d’ouverture LCR FO : fond d’œil IMC : indice de masse corporelle DGNO : dilatation gaine nerf optique FRCV : facteur de risque cardiovasculaire

## Discussion

Les résultats de la présente étude sont cohérents avec les données de la littérature, dans laquelle l’HII touche des femmes jeunes et obèses [6]. Au Niger, selon le rapport de l’enquête STEPS 2021, les prévalences du surpoids et l’obésité féminine étaient respectivement de 12,5 % et 6,3 % des cas [12].

Des études antérieures ont montré que l’obésité est un facteur de risque majeur pour l’HII [6,3]. Une étude cas-témoins multicentrique a démontré qu’un IMC élevé était associé à un risque progressivement plus élevé d’HII [3]. Dans notre série, cinq patientes étaient en surpoids ou obèses et ont rapporté que la symptomatologie avait commencé avec la prise de poids. Les autres patientes avaient un IMC normal. L’HII a été décrite chez des sujets avec un poids normal. Les céphalées sont les principales manifestations cliniques de l’HII et sont présentes dans environ 95 % des cas dès le début de la maladie [6]. Elles étaient présentes chez toutes les patientes de notre série. Ces céphalées sont inhabituelles et non spécifiques et ont la particularité d’être fréquentes, voire quotidiennes, dès l’apparition des symptômes. La plupart des patientes décrivent souvent une douleur pulsatile occipito-pariétale et souvent plus diffuse, mais ces maux de tête peuvent imiter une céphalée de tension ou une migraine. Une majoration des céphalées est classique lorsque la pression intracrânienne augmente, en particulier après une toux, une antéflexion ou en position allongée, ce qui peut conduire à des réveils « matinaux ». Les autres manifestations cliniques sont une baisse de la vision ou une vision floue, une diplopie due à la paralysie du nerf abducens, des photopsies, etc. [6]. Cependant, des études antérieures ont rapporté des cas d’HII sans œdème papillaire dans 5 à 15 % des cas [6,9].

L’imagerie cérébrale, en particulier l’IRM avec veinographie, montre une sténose du sinus veineux latéral dans environ 93 % des cas d’HII [4]. L’IRM peut révéler d’autres anomalies telles que l’aplatissement des globes, la tortuosité du nerf optique, la dilatation de la gaine, le vide sellaire et le déplacement inférieur du cervelet [8]. Les diagnostics différentiels sont les anomalies obstruant le drainage veineux cérébral (thrombose veineuse cérébrale, thrombose de la veine jugulaire, fistule artérioveineuse), le drainage du LCR entraînant une hydrocéphalie (méningite chronique, complication aiguë ou chronique d’une hémorragie méningée etc.), les processus expansifs intracrâniens (tumeurs, abcès etc.) et les causes iatrogènes liées à la prise de médicaments tels que les corticoïdes, fluoroquinolones, L-thyroxine, etc.

La prise en charge de l’HII a deux objectifs principaux, d’une part réduire ou éliminer les symptômes tels que les céphalées et, d’autre part préserver la fonction visuelle. L’acétazolamide est le principal traitement médicamenteux de l’HII. Il s’est avéré efficace pour améliorer les troubles visuels et la pression intracrânienne [10]. Il s’agit d’un médicament facilement accessible en termes de coût et disponibilité dans notre contexte, ce qui a facilité la prise en charge de nos patientes. La collaboration entre neurologue et ophtalmologue a permis un suivi régulier jusqu’à l’amélioration de l’acuité visuelle et la régression complète de l’œdème papillaire de nos patientes. L’obésité étant un facteur de risque majeur de l’HII [6,3], une perte de poids a également été recommandée, ce qui est facilité par le traitement à l’acétazolamide [10]. Des ponctions lombaires répétées peuvent être nécessaires chez les patients présentant un œdème papillaire sévère pour réduire rapidement la pression intracrânienne et, ainsi, améliorer les céphalées et les troubles visuels [15,16]. Chez les patients atteints de sténose du sinus latéral (SSL), le rôle causal de ce dernier dans la physiopathologie de l’HII reste controversé. Une étude portant sur cinq patients souffrant d’une HII associée à un syndrome de von Willebrand a montré une résolution clinique de leur HII sous traitement médical. Aucun des patients n’a présenté de changement évident de son syndrome de von Willebrand [1]. D’autres études ont montré que la pose de stent sur les SSL améliorait l’HII [2,13]. Au Niger, le *stenting* du SSL n’est pas encore réalisable malgré la présence d’une unité de neuroradiologie inter-ventionnelle (NRI) assez récente, dont la principale activité actuellement est la réalisation des angiographies cérébrales diagnostiques. Les stents adaptés dans notre contexte ne sont pas encore disponibles. Chez les patients dont la vision se détériore et qui souffrent de céphalées débilitantes ou de symptômes récurrents, des procédures moins invasives telles que la fenestration de la gaine du nerf optique, le cathéter lombopéritonéal ou la dérivation ventriculo-péritonéale peuvent être utilisées [14]. Le recours à ces méthodes n’a pas été nécessaire dans notre série de cas.

## Conclusion

L’HII est un trouble neurologique peu courant qui touche généralement les femmes jeunes et obèses. Son incidence augmente en raison de l’épidémie d’obésité. Il s’agit de la première série de cas nigériens d’HII. Nous avons retrouvé une présentation clinique habituelle comme décrite dans la littérature ainsi qu’une évolution favorable sous acétazolamide qui est facilement accessible à faible coût dans notre contexte. Cela a été réalisé grâce au plateau technique de notre hôpital relativement accessible à la population générale et surtout à une collaboration étroite multidisciplinaire entre médecins intervenants dans la prise en charge de cette pathologie, notamment ophtalmologues, neurologues et radiologues.

## Éthique

Notre étude a suivi les principes de la déclaration d’Helsinki. L’approbation d’un comité d’éthique n’a pas été nécessaire car il s’agissait d’une étude de soins de routine et les données ont été collectées de manière anonyme.

## Financement

L’étude n’a bénéficié d’aucun financement.

## Contributions des auteurs et autrices

Conception et design d’étude : ZM et SMA.

Collecte des données : ZM, SMA et MA.

Analyse et interprétation des données : ZM et MTD.

Recherches documentaires : ZM et MTD.

Rédaction du manuscrit : ZM et MTD.

Révision du manuscrit : ZM, SMA, AAB, FS, MA, HS, EA et MTD.

Tous les auteurs ont approuvé la version finale du manuscrit.

Garant de l’étude : ZM.

## Déclaration de liens d’intérêts

Aucun lien d’intérêt n’a été déclaré.
