# Supplementary material for: Idiopathic intracranial hypertension at the Niamey General Reference Hospital (Niger): a series of eight cases
Source: Med Trop Sante Int. 2026 Jan 13;6(1):mtsi.v6i1.2026.692. [Article in French] doi: 10.48327/mtsi.v6i1.2026.692 (PMC13216907; doi:10.48327/mtsi.v6i1.2026.692)
Supplement: Related texts

# Research article: Idiopathic intracranial hypertension at the Niamey General Reference Hospital (Niger): a series of eight cases

## Introduction

Idiopathic intracranial hypertension (IIH) typically affects young, obese women [9]. In Minnesota, USA, the incidence rate more than doubled from 1/100,000 in 1990 to 2.4/100,000 in 2014 due to increased rates of overweight and obesity [7]. IIH is a condition that has rarely been reported in sub-Saharan African countries. To our knowledge, only two cases have been reported in Mauritania, a West African country with a predominantly sub-Saharan population [10]. IIH is defined as headaches accompanied by papilledema and a cerebrospinal fluid opening pressure (CSF-OP) greater than 25 cm H₂O. This condition is characterized by the absence of hydrocephalus or brain mass syndrome on imaging and normal biochemical, bacteriological, and anatomopathological analyses of the CSF [5].

This study aims to report on the clinical and evolutionary characteristics of patients in Niger diagnosed with IIH in the neurology department of the Niamey General Reference Hospital.

## Methodology

The Niamey General Reference Hospital is one of Niger’s tertiary referral centers for managing neurological disorders. The neurology department, with its multidisciplinary team (neurologist, neurophysiologist, neurovascular specialist, physical therapist), offers advanced care in general neurology, epileptology, clinical neurophysiology, cerebrovascular disorders, etc.

We conducted a retrospective analysis of observations from patients in Niger diagnosed with IIH at our hospital between June 1, 2021, and May 31, 2023. Each patient underwent imaging of the cerebral parenchyma (low-field MRI: 0.35 Tesla or 16-slice CT scan) and intracranial vessels (veins and arteries), as well as a cerebrospinal fluid study (cytorachia, proteinorachia, glycora-chia, culture, and search for atypical cells), and laboratory tests to detect biological inflammatory syndromes and quantify D-dimers. The CSF-OP opening pressure was measured in each patient using a manometer attached to the needle while the patient was lying in the left lateral decubitus position. All patients included in the study met the International Headache Society (IHS) criteria for IIH (latest version: ICHD-3B) [5].

The following information was collected from each patient’s medical records: age, sex, body mass index (BMI), medical history, cardiovascular risk factors, results of an ophthalmological examination, results of brain and vascular imaging, CSFOP results, and results of a CSF analysis.

This study followed the principles of the Declaration of Helsinki. Approval from an ethics committee was not required, as this was a routine care study, and the data were collected anonymously. All patients gave informed consent to participate in the study.

Patient characteristics were expressed as percentages for categorical variables and as medians with interquartile ranges (IQRs) for continuous variables. The Shapiro-Wilk test was used to assess the normality of all quantitative measures. Statistical analyses were performed using IBM SPSS Statistics for Windows, Version 25.0 (Armonk, NY).

## Results

During the study period, 2,700 female patients were seen for consultations. Of those patients, eight were diagnosed with IIH, representing a hospital prevalence of 3 per 1,000 (3‰). Table I details the characteristics of the eight patients. All of the patients were women with a median age of 29.5 years (interquartile range [IQR]: 21.5–37.75) and a median body mass index (BMI) of 30.75 kg/m² (IQR: 26–32). None of the patients had a medical history of primary headaches (e.g., migraines or tension headaches) or cardiovascular risk factors other than obesity. None were exposed to infectious diseases, particularly *Mycobacterium tuberculosis*. Six patients were referred by ophthalmologists for a neurological consultation due to headaches with subacute onset and bilateral papilledema. Bilateral papilledema was observed in all patients in our study (Fig. 1). A neurological examination revealed binocular diplopia with abducens nerve palsy (nerve VI: external oculomotor nerve) in five patients. The rest of the clinical examination (general and neurological) was normal. Seven patients underwent brain MRI, and only one underwent a brain CT scan and cerebral angiography. Brain imaging revealed no parenchymal abnormalities or lateral sinus stenosis. One patient showed dilation of the optic nerve sheath on MRI T2 axial and coronal sequences (Fig. 2). The median CSF pressure on opening was 30 cm H₂O (IQR: 28.35–35).

**Table I:** Clinical and radiological characteristics of 8 cases of intracranial hypertension at the Niamey General Reference Hospital (2021-2023)

Variable	Patient 1	Patient 2	Patient 3	Patient 4	Patient 5	Patient 6	Patient 7	Patient 8
Age (years)	31	28	18	55	23	21	31	40
BMI (kg/m^2^)	28,2	30,2	25,3	32,1	35	31,3	20,2	32
Medical history & CVRF	No	No	No	No	No	No	No	No
LVVA	9/10	5/10	8/10	7/10	-	-	4/10	7/10
RVVA	7/10	6/10	8/10	8/10	-	-	5/10	7/10
DFE	BP	BP	BP	BP	BP	BP	BP	BP
Nerve VI palsy	Yes	No	Yes	Yes	Yes	No	Yes	No
LSS	No	No	No	No	No	No	No	No
ONSD	No	No	No	No	No	Yes	No	No
CSF-OP (cmH_2_O)	37	27	35	29	35	28	30	30
CSF analysis	Normal	Normal	Normal	Normal	Normal	Normal	Normal	Normal

BP: bilateral papilledema LVVA: center visual acuity RVVVA: right visual acuity medical history LSS: Lateral sinus stenosis CSF-OP DFE: dilated fundus examination BMI: Body mass index ONSD: optic nerve sheath dilation CVRF: Cardiovascular risk factor

**Figure 1:** Papilledema

**Figure 2:** Axial T2-weighted MRI of the orbit and brain, showing dilation of the right optic nerve sheath

All patients were treated with acetazolamide (250 mg every 8 hours, for at least 3 months, with gradual reduction and monitoring of sodium and potassium levels.) and recovered full vision and normal fundus appearance after two to three months of treatment. Treatment was then discontinued. No patients experienced any side effects. Non-drug treatment consisted of reducing CSF pressure by removing 40 to 50 mL of CSF during a lumbar puncture and following an unsaturated, weight-loss diet. Six months after stopping acetazolamide, one patient relapsed, requiring the reintroduction of long-term treatment.

## Discussion

The results of this study are consistent with previous studies, which indicate that IIH affects young, obese women [6]. According to the 2021 STEPS survey report, the prevalence of overweight and obesity in women in Niger was 12.5% and 6.3%, respectively [12].

Previous studies have shown that obesity is a major risk factor for IIH [6,3]. A multicenter case-control study demonstrated that a high BMI is associated with an increasingly higher risk of IIH [3]. In our study, five patients were overweight or obese and reported that their symptoms began with weight gain. The other patients had a normal BMI. IIH has been described in subjects of normal weight. Headaches are the main clinical manifestation of IIH, present in approximately 95% of cases from the onset of the disease [6]. They were present in all patients in our series. These headaches are unusual and nonspecific, characterized by their frequency—even daily—from the onset of symptoms. Patients often describe pulsating occipito-parietal pain, which is usually more diffuse; however, these headaches can mimic tension headaches or migraines. An increase in headache frequency is typical when intracranial pressure rises, particularly after coughing, bending forward, or lying down. This can lead to “early morning” awakenings. Other manifestations include decreased or blurred vision, diplopia due to abducens nerve palsy, and photopsia. [6]. However, previous studies have reported cases of IIH without papilledema in 5-15% of patients [6,9]. Brain imaging, particularly magnetic resonance imaging (MRI) with venography, reveals lateral sinus stenosis in approximately 93% of IIH cases [4]. An MRI may also reveal additional abnormalities, including flattening of the globes, tortuosity of the optic nerve, dilation of the sheath, an empty sella turcica, and inferior displacement of the cerebellum [8]. Differential diagnoses include obstructions of cerebral venous drainage (e.g., cerebral or jugular vein thrombosis, arteriovenous fistulas), obstructions of CSF drainage leading to hydrocephalus (e.g., chronic meningitis, complications of subarachnoid hemorrhage), and intracranial expansive processes (e.g., tumors, abscesses). Iatrogenic causes may be related to medications such as corticosteroids, fluoroquinolones, and L-thyroxine.

The management of IIH has two main objectives: reducing or eliminating symptoms, such as headaches, and preserving visual function. Acetazolamide is the primary drug treatment for IIH. It has been shown to effectively improve visual disturbances and intracranial pressure [10]. It is easily accessible in terms of cost and availability, which has facilitated management of our patients. Collaboration between neurologists and ophthalmologists enabled regular follow-up until our patients’ visual acuity improved and their papilledema completely regressed. Since obesity is a major risk factor for IIH [6,3], weight loss has also been recommended and is facilitated by acetazolamide treatment [10]. Repeated lumbar punctures may be necessary for patients with severe papilledema to rapidly reduce intracranial pressure, thereby improving headaches and visual disturbances [15,16]. In patients with lateral sinus stenosis (LSS), the causal role of LSS in the pathophysiology of IIH remains controversial. A study of five patients with IIH associated with von Willebrand syndrome showed that their IIH resolved with medical treatment. None of the patients showed any obvious change in von Willebrand syndrome [1]. Other studies have shown that stenting LSS improves IIH [2,13]. In Niger, despite the presence of a relatively new interventional neuroradiology (INR) unit whose main activity is currently diagnostic cerebral angiography, LSS stenting is not yet feasible. Stents suitable for this context are not yet available. For patients experiencing deteriorating vision, debilitating headaches, or recurrent symptoms, less invasive procedures, such as optic nerve sheath fenestration, lumboperitoneal catheterization, or ventriculoperitoneal shunting, may be employed [14]. These methods were not necessary in our case series.

## Conclusion

IIH is a rare neurological disorder that primarily affects young, obese women. Its incidence is increasing due to the obesity epidemic. This is the first series of cases in Niger of IIH. We observed the typical clinical presentation described in the literature, as well as a favorable outcome with acetazolamide, which is readily available and inexpensive in our setting. This favorable outcome was achieved thanks to our hospital’s technical facilities, which are relatively accessible to the general population. Most importantly, it was achieved through close multidisciplinary collaboration between the physicians involved in managing this condition, including ophthalmologists, neurologists, and radiologists.

## Ethics

Our study adhered to the principles of the Declaration of Helsinki. Approval from an ethics committee was unnecessary because this was a routine care study, and the data were collected anonymously.

## Funding

The study did not receive any funding.

## Authors’ contribution

Study conception and design: ZM and SMA

Data collection: ZM, SMA, and MA.

Data analysis and interpretation: ZM and MTD

Literature review: ZM and MTD

Manuscript writing: ZM and MTD

Manuscript revision: ZM, SMA, AAB, FS, MA, HS, EA and MTD.

All authors approved the final version of the manuscript.

Study sponsor: ZM.

## Conflicts of interest

The authors declare no conflicts of interest
